# Infantile epididymitis with calcification

**DOI:** 10.4103/0971-9261.42570

**Published:** 2008

**Authors:** Katsumi Muramori, Koji Nagata, Noritoshi Handa

**Affiliations:** Division of Pediatric Surgery, Oita Prefectural Hospital, 476 Bunyou Oita, Japan

**Keywords:** Infantile epididymitis calcification

## Abstract

A 1-month-old infant presented with a case of calcifying chronic epididymitis. Differential diagnosis was made from a testicular torsion and neoplasm. Serial ultrasound examination revealed a calcified lesion adjacent to the normal testis, thereby avoiding an unnecessary orchiectomy. Infantile epididymitis has been thought to be rare; however, it is occasionally encountered in the literature and calcification with chronic epididymitis in an infant has not been previously reported. On the other hand, an infant with scrotal calcification should be suspected of neoplasm. However, the tumor markers α-fetoprotein (AFP) and Human Chorionic Gonadotropin β (HCG β) were within the physiological range. Therefore, a diagnosis must be carefully made to avoid an unnecessary orchiectomy.

## INTRODUCTION

Pediatric epididymitis had been diagnosed in 35% children (84 cases within 2 years) presenting with acute scrotum,[1] and a recent report showed an incidence of around 5-40 cases of acute epididymitis per year.[2] However, infantile epididymitis has been considered rare an incidence of approximately 2-7% incidence reported in infancy.[1,3] On the other hand, infantile chronic epididymitis is extremely rare in the literature and it is difficult to differentiate from neonatal testicular torsion or a tumor in a a painless intratesticular mass.

Although a diagnosis can be made by a scrotal ultrasound examination, in the case of chronic epididymitis, an accurate diagnosis remains difficult due to the absence of acute inflammatory symptoms such as pain, redness and swelling. Furthermore, a case in which only testicular enlargement is prominent may require an inguinal exploration with an orchiectomy for a diagnostic and therapeutic approach in case of a suspected malignancy.

In this report, a case of infantile chronic epididymitis, presenting with scrotal calcification which required a differential diagnosis from a testicular torsion and neoplasm, was demonstrated.

## CASE REPORT

A 1-month-old baby was admitted for right scrotal swelling and enlargement of the right testis. The infant was born by transvaginal delivery with a birth weight of 2230 g and was admitted to another hospital because of hypoglycemia. On day 1, leukocytopenia: 2800/mm^3^ and thrombocytopenia: 10.4 × 10^4^/mm^3^ were reported and an antibiotic was administered with a diagnosis of infection of unknown origin. On day 6, a right scrotal swelling was noted, and antibiotics were administered for another 5 days. Following the discharge from the hospital, the right scrotal swelling persisted, and the patient was admitted to our hospital for an examination of scrotal enlargement.

**Figure 1:**
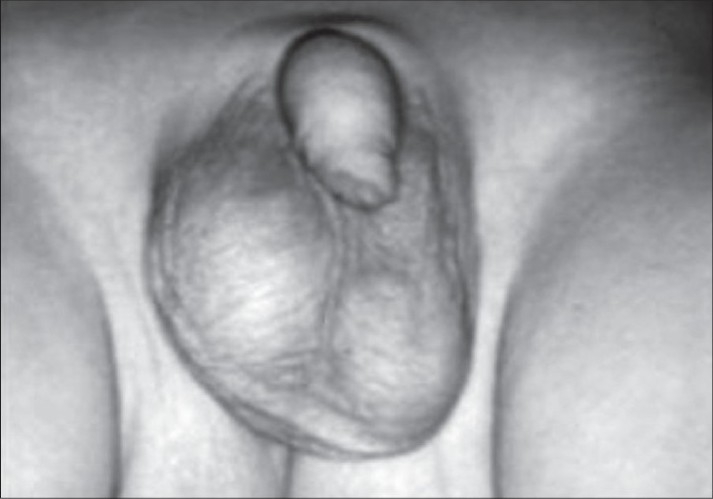
Painless mass (2.5 cm in size) in the major axis was palpable in the right scrotum

**Figure 2:**
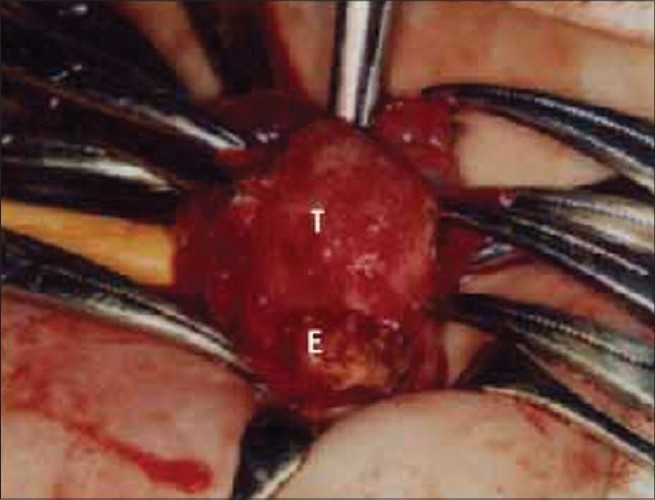
Right testis (T) was encapsulated by fine granules and adjacent epididymis (E) was replaced by calcified mass

In the right swollen scrotum, a hard mass with a size of approximately 2.5 × 1.5 cm was palpated without tenderness, [Figure 1] and an ultrasonic examination revealed a mass (2.5 cm in size) with multiple macular hyperechoic lesions, which was considered as calcification. However, a normal testicular echo was not determined. On examination, a neonatal testicular torsion was primarily suspected. However, a differential diagnosis was necessary for the testicular tumors. The level of tumor markers were as follows: AFP: 13,764 ng/ml and HCG-β < 0.1 ng/ml. AFP was slightly higher than the level appropriate for the patients age. However, the physiological decline of AFP with no increase in the size of the mass was observed. A sequential ultrasound examination revealed that the calcification was located primarily in the epididymis with the appearance of an adjacent normal testicular echo. The patient had right inguinal hernia after 2 months. He underwent a repair of the inguinal hernia and biopsy of the calcified mass simultaneously at 6 months. The surface of the testis was granular, and both testes and the adjacent calcifying lesion were partially resected [Figure 2]. Pathological findings showed chronic epididymitis with calcification and a normal testis.

## DISCUSSION

Scrotal calcification may occur in association with testicular tumor in children; however, in the radiological review, extratesticular calcification was mentioned to be more frequently encountered and usually related to the previous inflammatory disease of the epididymis.[4] In the literature, infantile chronic epididymitis is extremely rare, and a case presenting as a painless intrascrotal mass should be differentiated from a neoplasm and old torsion. Kiss *et al*. reported that asymptomatic intrascrotal masses were most frequently (63.2%) encountered in young boys, whose age was less than 1 year old, and surgical exploration revealed that 23% of cases were diagnosed as epididymitis in which two cases of dystrophic calcification were determined as a result of chronic epididymitis.[5]

In acute epididymitis, a differential diagnosis from testicular torsion should be made using Doppler flowmetry or testicular scintigraphy;[6] however, color Doppler ultrasonography is recommended because of its reproducibility and prompt dagnosis and it also reduces the need for surgical exploration.[7] On the other hand, chronic epididymitis may present as a painless intrascrotal mass, and therefore, it is very significant to distinguish this from a malignant tumor. In our case, sequential ultrasonography revealed that the mass with calcification appeared not to be in growth and contributing to chronic epididymitis; incidentally, this was confirmed during the repair of the right inguinal hernia and an unnecessary orchidectomy was avoided.

Urogenital anomalies were highly recognized in paediatric epididymitis with 47% incidence in prepubertal child and 75% in infantile cases;[8] however, a recent report showed anatomic urinary anomalies in 18% of epididymitis in children and infants.[9] Haecker and associates diagnosed only one boy with urinary malformation out of 49 children who presented with acute epididymitis.[3] Rare cases complicating epididymitis at infancy were also reported to be caused by urogenital anomalies associated with anorectal malformation.[10] To these underlying urogenital anomalies, a positive urine culture has also been reported to be highly attributed.[8] However, a recent report by Cappele *et al*. showed that there was no significant relationship observed between the prognostic factors related to the recurrence and underlying anomalies and concluded that further urogenital exploration was recommended in patients presenting with recurrent epididymitis.[9]

In contrast, the cause of epididymitis without urogenital anomalies was categorized as follows: viral or bacterial infection, systemic disease such as sarcoidosis, Kawasaki disease or Schönlein-Henoch purpura, trauma, anti-arrhythmic drug amiodarone and clean intermittent catheterization.[2] In our case, no other anomalies were found using genitourinary imaging, and systemic infection during the neonatal period was suspected to be a cause.

In an infant with scrotal calcification, an old testicular torsion[11] or neoplasm should be primarily suspected; however, we believe that it is also important to take an infantile epididymitis into consideration and that an infantile scrotal calcification may not immediately be an indication for orchidectomy.
